# Tumor Segmentation on PSMA PET/CT Predicts Survival in Biochemical Recurrence of Prostate Cancer: A Retrospective Study Using [^68^Ga]Ga-PSMA-11 and [^18^F]-PSMA-1007

**DOI:** 10.3390/cancers17132249

**Published:** 2025-07-04

**Authors:** Ken Kudura, Yves Schaulin, Arnoud J. Templeton, Tobias Zellweger, Wolfgang Harms, Raphael Georis, Michael C. Kreissl, Robert Foerster

**Affiliations:** 1 Department of Nuclear Medicine, University Hospital CHU UCL Godinne, 5530 Yvoir, Belgium; 2 Faculté de Médecine, Université Catholique de Louvain UCLouvain, 1348 Louvain, Belgium; 3 Faculty of Medicine, University of Basel, 4001 Basel, Switzerland; 4 Sankt Clara Research, 4058 Basel, Switzerland; 5 Department of Urology, Sankt Clara Hospital, 4058 Basel, Switzerland; 6 Department of Radiation Oncology, Sankt Clara Hospital, 4058 Basel, Switzerland; 7 Department of Biostatistics, University Hospital CHU UCL Godinne, 5530 Yvoir, Belgium; 8 Division of Nuclear Medicine, Department of Radiology and Nuclear Medicine, University Hospital Magdeburg, 39120 Magdeburg, Germany; 9 Department of Radiation Oncology, Cantonal Hospital Winterthur, 8400 Winterthur, Switzerland; robert.foerster@ksw.ch; 10 University Hospital, University of Bern, 3012 Bern, Switzerland

**Keywords:** [^68^Ga]Ga-PSMA-11, [^18^F]-PSMA-1007, PET/CT, prostate cancer, biochemical recurrence, tumor segmentation, outcome prediction

## Abstract

**Background:** PSMA PET/CT imaging has become a cornerstone in the management of prostate cancer, particularly in the setting of biochemical recurrence (BCR). While semi-quantitative parameters such as SUVmean have been evaluated as prognostic biomarkers in metastatic castration-resistant prostate cancer (mCRPC), particularly after the VISION trial, the prognostic role of volumetric measures such as Total Molecular Volume (TMV) remain largely unexplored, especially in earlier stages of the disease such as in biochemical recurrence following primary treatment. **Methods:** This retrospective monocentric study included 84 patients with BCR who underwent PSMA PET/CT imaging between 2020 and 2021 using either [^68^Ga]Ga-PSMA-11(Ga-PSMA) or [^18^F]-PSMA-1007 (F-PSMA) as tracers. Total tumor burden was assessed through manual 3D segmentation to derive whole-body Total Molecular Volume (wb TMV) and Total Lesion PSMA (wb TL-PSMA). Clinical and imaging variables were correlated with overall survival (OS) and progression-free survival (PFS) using Cox regression models. Kaplan–Meier analyses were performed based on wb TMV and thresholds determined by the Youden index. **Results:** A PSMA PET/CT correlation for BCR was identified in 69% of patients, with comparable detection rates between tracers (Ga-PSMA 67% vs. F-PSMA 63%, *p* = 0.7). A higher wb TMV was significantly associated with worse OS (HR 2.20, *p* < 0.001) and PFS (HR 2.01, *p* < 0.001) in univariable analyses. In multivariable models, log_2_(wb TMV) remained an independent prognostic factor for PFS (HR 1.78, *p* = 0.005). Patients with log_2_(wb TMV) > 2.87 exhibited significantly poorer survival outcomes. A PSA at diagnosis > 17 ng/mL also predicted shorter PFS. **Conclusions:** Tumor segmentation from PSMA PET/CT imaging provides powerful prognostic information in patients with biochemical recurrence of prostate cancer, independently of the tracer used. The wb TMV represents a promising volumetric biomarker for future risk stratification and therapeutic decision-making, particularly in earlier stages of prostate cancer progression, where predictive imaging biomarkers remain largely undefined.

## Simple Summary

This retrospective study evaluated the prognostic value of tumor volume segmentation on PSMA PET/CT in men with a biochemical recurrence (BCR) of prostate cancer. A total of 84 patients underwent either [^68^Ga]Ga-PSMA-11 or [^18^F]F-PSMA-1007 PET/CT. Manual 3D segmentation was performed to extract semi-quantitative parameters, including whole-body Total Molecular Volume (wb TMV) and Total Lesion PSMA (wb TL-PSMA). Cox regression models were used to assess associations with overall survival (OS) and progression-free survival (PFS). Detection rates were comparable between tracers (69% positive scans). A higher wb TMV was significantly associated with poorer OS and PFS. Log_2_(wb TMV) showed the highest area under the curve (AUC = 0.81 for OS) compared to other parameters, outperforming PSA at diagnosis and wb TL-PSMA. Despite a limited sample size, this study provides novel evidence that volumetric assessment via PSMA PET/CT can serve as a reliable imaging biomarker in early prostate cancer relapse. These findings support the incorporation of tumor volume quantification into clinical workflows to improve patient risk stratification and treatment selection. Future prospective multicenter studies are warranted to validate these results.

## 1. Introduction

Prostate cancer remains the most common malignant neoplasm in men and represents the second leading cause of cancer-related mortality among men in Switzerland, with approximately 7800 new diagnoses and 1350 deaths annually [1]. Globally, prostate cancer remains a major cause of cancer-related morbidity and mortality [2,3,4]. For non-metastatic disease, curative treatment options such as radical prostatectomy or radiotherapy combined with androgen deprivation therapy (ADT) are available [5]. In the metastatic setting, the disease is considered palliative, with ADT serving as the cornerstone of therapy. Over recent years, several large, randomized Phase III trials have demonstrated significant improvements in prognosis (overall survival) through the early addition of docetaxel chemotherapy [6,7], and novel androgen receptor pathway inhibitors such as abiraterone [8,9], apalutamide [10], darolutamide [11], enzalutamide [12], or combinations thereof [13,14].

PSMA PET/CT is a highly sensitive and specific hybrid imaging modality that combines molecular imaging of prostate-specific membrane antigen (PSMA) expression using positron emission tomography (PET) with detailed anatomical information from computed tomography (CT). Its clinical utility spans several key stages of prostate cancer management. First, PSMA PET/CT is recommended for initial staging in patients with intermediate- or high-risk disease, providing crucial information for risk stratification and therapeutic decision-making. Second, it plays a central role in the detection of disease relapse in patients with suspected biochemical recurrence after definitive local therapy, facilitating early diagnosis and tailored management [15,16,17,18,19].

Third, in the advanced setting of metastatic castration-resistant prostate cancer (mCRPC), PSMA PET/CT is essential for selecting patients eligible for targeted metabolic treatments such as radioligand therapy (RLT) using [^177^Lu] labelled compounds [20,21,22,23,24]. Since the establishment of RLT as a third-line treatment for mCRPC based on pivotal studies including the VISION trial [22], efforts have been undertaken to identify predictive biomarkers of response and survival. The mean standardized uptake value (SUVmean) has been proposed as an important prognostic factor [25,26,27]. However, tumor burden assessment still heavily relies on visual and semi-quantitative analyses, limiting the predictive power of clinical assessments. Tumor segmentation—precise quantification of tumor lesions to determine Total Molecular Volume—could offer an objective and reproducible alternative to evaluate disease extent and optimize patient stratification.

While the literature has primarily focused on the prognostic value of the SUVmean and other semi-quantitative parameters in advanced disease settings, particularly following the VISION study in the context of RLT, few studies have addressed the role of tumor segmentation in cases of biochemical recurrence [28,29]. Accurate segmentation of lesions detected by PSMA PET/CT may enhance long-term survival prediction, refine therapeutic indications, and improve patient management, including eligibility selection for RLT.

The aim of this study was to investigate the clinical value of tumor segmentation in PSMA PET/CT imaging for the suspected biochemical recurrence of prostate cancer and to compare detection rates between two different radiotracers. Additionally, we sought to explore the prognostic value of clinical and imaging variables.

## 2. Methods

### 2.1. Study Design and Patient Population

We retrospectively included patients who, between 2020 and 2021, experienced a biochemical recurrence (BCR), defined as a rise in Prostate-Specific Antigen (PSA), following one or more treatments for localized prostate cancer at our DKG-certified Prostate Cancer Center, and who underwent PSMA PET/CT imaging. BCR was defined according to standard criteria, based on primary treatment modality. For patients treated with radical prostatectomy, BCR was defined as a PSA level ≥ 0.2 ng/mL, confirmed by a second measurement; for patients treated with radiotherapy, BCR was defined as a PSA rise ≥ 2.0 ng/mL above the post-treatment nadir. Patients were identified through the uro-oncological tumor center database.

This retrospective, monocentric study was approved by the Ethics Committee for Northwest and Central Switzerland (EKNZ 2022-01414) and conducted in accordance with Good Clinical Practice (GCP) guidelines and the Declaration of Helsinki. Informed consent for data usage (“General consent”) was obtained from all included patients [30,31].

### 2.2. Data Collection

Clinical (Section 2.2.1) and imaging data (Section 2.2.2) were extracted from medical reports, tumor board recommendations, and imaging findings and were entered into a predefined database. Follow-up data were obtained from physician reports, tumor board recommendations, or laboratory results. For patients followed up externally (*N* = 20), the treating general practitioner or urologist was contacted.

#### 2.2.1. Clinical Data

The following clinical parameters were collected: age at BCR, date of initial prostate cancer diagnosis, PSA at diagnosis, Gleason score, TNM stage at diagnosis, treatment modality, date of definitive therapy, date of BCR, PSA value at BCR, date of hybrid imaging (PSMA PET/CT), and PSMA tracer used.

#### 2.2.2. Imaging Data

Semi-quantitative imaging parameters extracted from PSMA PET/CT scans included the following:

- **SUVmax** (maximum standardized uptake value): the highest single voxel SUV value within a region of interest (ROI).
- **SUVmean** (mean standardized uptake value): the arithmetic mean of the standardized uptake values of all voxels included in each ROI, which in this study was segmented using a relative threshold of 40% of the lesion’s SUVmax, as previously described in the study by Gafita et al. [32].
- **Total Molecular Volume (TMV)**: the sum of all voxels within each ROI exhibiting uptake ≥ 40% of the lesion’s SUVmax, as previously described in the study by Gafita et al. [32]. The whole-body Total Molecular Volume (wb TMV) was defined as the sum of TMVs of all tumor manifestations identified on PSMA-PET per patient.
- **Total Lesion PSMA (TL-PSMA)**: the product of the TMV and the corresponding mean standardized uptake value (SUVmean) within that volume. The whole-body Total Lesion PSMA (wb TL-PSMA) was defined as the sum of TMVs of all tumor manifestations identified on PSMA-PET per patient.

#### 2.2.3. Qualitative Analysis

Qualitative assessment of PSMA PET/CT scans was performed by two board-certified dual-specialty physicians (radiology and nuclear medicine) following a double-reading procedure to ensure quality control. Findings were categorized into three groups:

1. **Positive scan**: clear morphological correlation for BCR.
2. **Negative scan**: no pathological PSMA uptake.
3. **Indeterminate scan**: no clear assignment to either positive or negative findings.

#### 2.2.4. Quantitative Analysis

Quantitative analysis focused on PSMA-avid lesions identified as malignant or indeterminate by clinical interpretation. Lesions were manually delineated using a 3D contouring tool (advanced workstation AW by General Electric, Milwaukee, WI, USA). PSMA PET/CT datasets were reviewed in sagittal, transverse, and coronal planes. CT images could be reviewed independently or fused with PET images for precise lesion localization. Care was taken to delineate lesions as accurately as possible while avoiding physiological uptakes or unrelated PSMA activity. Contrast medium was not used systematically, but was used in the majority of the cases. Manual contouring was feasible even for small lesions. The smallest measurable lesion volume was defined by the ROI used as 1 mL. In the case of small lesions or lesions with low contrast against background uptake, manual refinement was performed to exclude physiological uptake or adjacent structures. To minimize inter-observer variability, segmentations were reviewed independently by two board-certified physicians, and discrepancies were resolved by consensus.

### 2.3. Radiotracers and Image Acquisition

Two radiotracers were used, [^68^Ga]Ga-PSMA-11 and [^18^F]-PSMA-1007, both administered via intravenous bolus injection.

PSMA PET/CT images were acquired in clinical routine, following the standard clinical protocol. Patients received either an intravenous injection of Ga-PSMA-11 (at a dose of 2–3 MBq/kg) or F18-PSMA (at a dose of 3–4 MBq/kg). PET acquisition was performed 60 min post-injection, routinely from the skull base to mid-thigh and in selected cases extended to the feet. A low-dose CT scan was acquired for attenuation correction and anatomical localization, followed by a 3D PET acquisition with a duration of 2.5 min per bed position. Images were reconstructed using a vendor-specific iterative algorithm (OSEM, 3 iterations, 16 subsets, time-of-flight and resolution modeling).

### 2.4. Outcomes

The primary outcome was the detection rate of tumor manifestations according to the PSMA PET/CT scans using [^68^Ga]Ga-PSMA-11 and [^18^F]-PSMA-1007 tracers.

The secondary outcome was to identify clinical and imaging biomarkers associated with overall survival (OS) and progression-free survival (PFS).

### 2.5. Statistical Analysis

First, descriptive statistics were performed, presenting absolute numbers and relative percentages for the entire cohort and according to the radiotracer used. The frequency of pathological findings (categories 1–3) was analyzed. For pathological findings (categories 1 and 3), the number and anatomical localization of detected lesions were assessed across six predefined regions corresponding to typical recurrence patterns. Lesions outside these areas were grouped under “other”.

Categorical variables were compared using Fisher’s exact test or a Chi-square test, and continuous variables using the Wilcoxon–Mann–Whitney test. For survival analysis, univariable Cox regression analyses were initially conducted for various clinical and imaging factors, followed by multivariable modeling for significant variables. Kaplan–Meier curves were generated to visualize survival times, calculated from the date of PSMA PET/CT to death or censored at last follow-up.

The wb TL-PSMA values were divided by 100 for interpretability; wb TMV values underwent a logarithmic transformation (log2) when non-normally distributed. Cut-off values for continuous variables were determined using the Youden index from ROC curve analyses [33]. A two-sided *p*-value < 0.05 was considered statistically significant; no corrections were made for multiple comparisons.

## 3. Results

### 3.1. Patient Population

**Figure 1:**
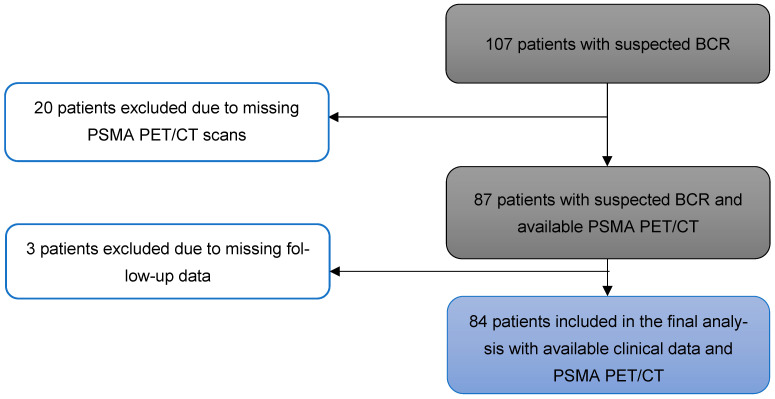
Flow chart for patient inclusion (N = 84 patients).

Out of 107 patients with biochemical recurrence, 20 were excluded due to the absence of PSMA PET/CT imaging, and an additional 3 were excluded due to missing follow-up data. Thus, a total of 84 patients were included in the final analysis (Figure 1).

The majority of patients (*N* = 60, 71%) underwent Ga-PSMA PET/CT, while the remaining (*N* = 24, 29%) underwent F-PSMA PET/CT.

**Figure 2:**
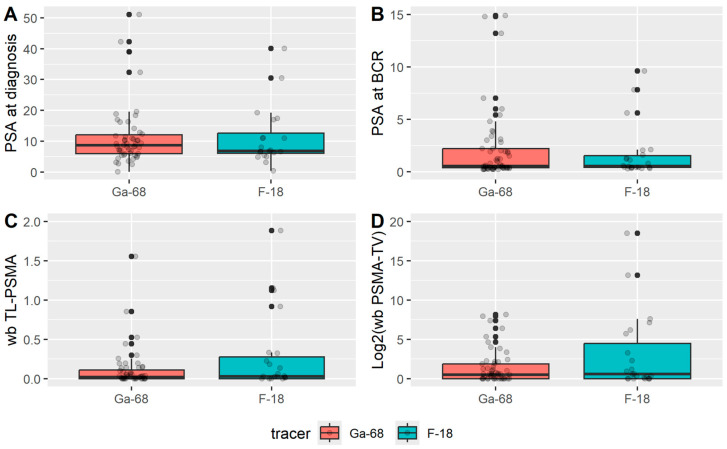
Box plots showing the distribution of (**A**) PSA at diagnosis, (**B**) PSA at BCR, (**C**) wb TL-PSMA, and (**D**) Log2(wb PSMA-TV) according to the tracer used.

Patient characteristics are summarized in Table 1. The median age was 72 years. Patients scanned with F-PSMA were slightly older, but this difference was not statistically significant. The median body mass index (BMI) was 25.2 kg/m^2^ and was comparable between groups. No significant differences were observed in PSA levels at diagnosis or at biochemical recurrence between the two tracer groups, suggesting comparable baseline tumor activity (Table 1 and Figure 2).

### 3.2. PSMA PET/CT Findings

#### 3.2.1. Pathological PSMA PET/CT Findings (Category 1)

Among the 84 included patients, 58 (69%) had a positive PSMA PET/CT scan (Category 1), meaning a clear imaging correlation for biochemical recurrence.

The majority of patients with pathological findings (66%) showed a localized recurrence, with comparable detection rates between Ga-PSMA and F-PSMA (67% vs. 63%, *p* = 0.7) (Table 2).

In terms of lesion distribution according to predefined anatomical regions, local recurrences (prostatic fossa) were the most common (59%), followed by pelvic nodal recurrences (38%). Bone metastases represented the third most frequent site (26%).

While 23% of patients imaged with Ga-PSMA showed bone metastases, 32% of those scanned with F-PSMA showed such lesions. No statistically significant differences were observed between tracers regarding lesion localization.

#### 3.2.2. Indeterminate PSMA PET/CT Findings (Category 2)

In 14 out of 84 PSMA PET/CT scans (17%), findings were classified as indeterminate (Category 2). Comparing tracers, no statistically significant differences were observed overall (Table 3). Unclear lesions located in the prostatic fossa were found in 29% of indeterminate scans. Half of the indeterminate lesions were located in the pelvis, all among patients scanned with Ga-PSMA. Interestingly, indeterminate bone lesions were more frequent with F-PSMA.

Since detection rates were comparable between the two tracers, all further analyses were conducted for the combined study population.

### 3.3. Survival Analysis Using Cox Regression Models

#### 3.3.1. Univariable Cox Model for Overall Survival (OS)

During the observation period, 11 of the 84 patients (13%) died.

In univariable Cox regression analysis, a higher wb TL-PSMA, log_2_(wb TMV), and PSA at biochemical recurrence were associated with poorer prognosis (hazard ratio [HR] > 1), whereas SUVmax, SUVmean, and PSA at initial diagnosis were not significantly associated (Table 4).

#### 3.3.2. Multivariable Cox Model for Overall Survival (OS)

Variables with significance in univariable analyses (wb TL-PSMA, log_2_(wb TMV), PSA at BCR) were included in a multivariable Cox model. Here, only log_2_(wb TMV) showed a borderline significant association with prognosis (Table 5).

#### 3.3.3. Risk Stratification (OS) Based on log_2_(wb TMV)

Using the Youden index, an optimal log_2_(wb TMV) cut-off of 2.87 was calculated.

**Figure 3:**
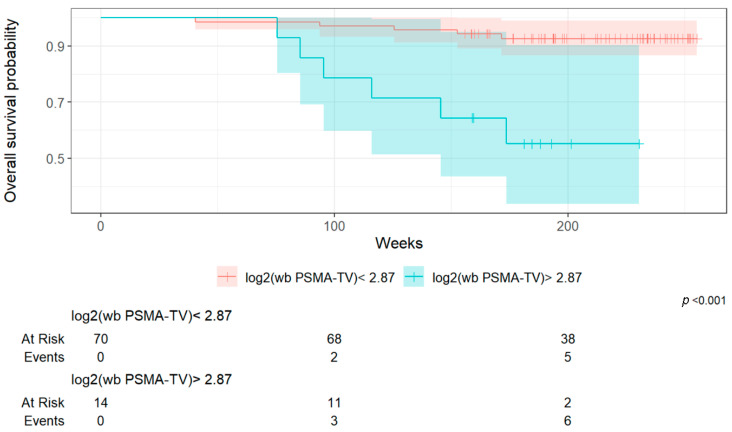
Kaplan–Meier survival curves (OS) according to log_2_(wb TMV) cut-off (2.87).

Patients with log_2_(wb TMV) > 2.87 had significantly worse survival outcomes compared to those with log_2_(wb TMV) ≤ 2.87 (Figure 3).

#### 3.3.4. Univariable Cox Model for Progression-Free Survival (PFS)

In addition to overall survival (OS), a further analysis was performed to evaluate factors associated with progression-free survival (PFS).

The univariable Cox regression analysis identified log_2_(wb TMV), PSA at initial diagnosis, and PSA at biochemical recurrence as being associated with a higher risk of progression (hazard ratio [HR] > 1), whereas SUVmax and SUVmean were not significantly associated (Table 6).

#### 3.3.5. Multivariable Cox Model for Progression-Free Survival (PFS)

Variables with significance in univariable analyses, log_2_(wb TMV) and PSA at initial diagnosis, and at biochemical recurrence, were included in a multivariable Cox model.

Here, log_2_(wb TMV) and PSA at initial diagnosis showed a significant association with risk of progression (Table 7).

#### 3.3.6. Risk Stratification (PFS) Based on log_2_(wb TMV) and PSA at Diagnosis

Using the Youden index method, cut-off values were determined for the two relevant parameters, log_2_(wb TMV) and PSA at diagnosis, allowing for the stratification of the patient population into two distinct risk groups for each parameter.

**Figure 4:**
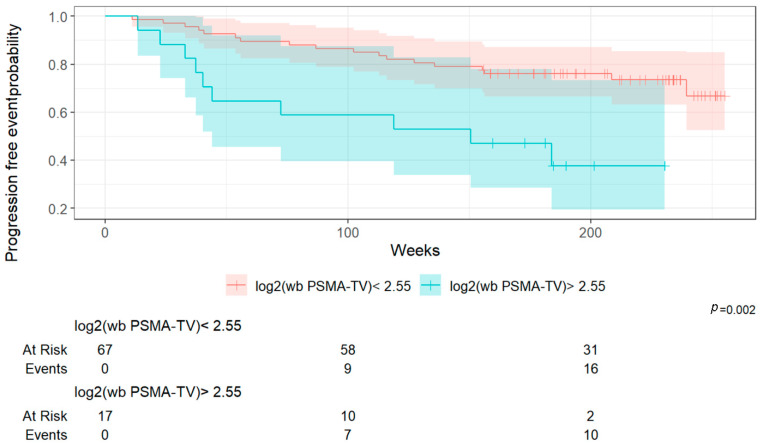
Kaplan–Meier survival curves (PFS) according to log_2_(wb TMV) cut-off (2.55).

**Figure 5:**
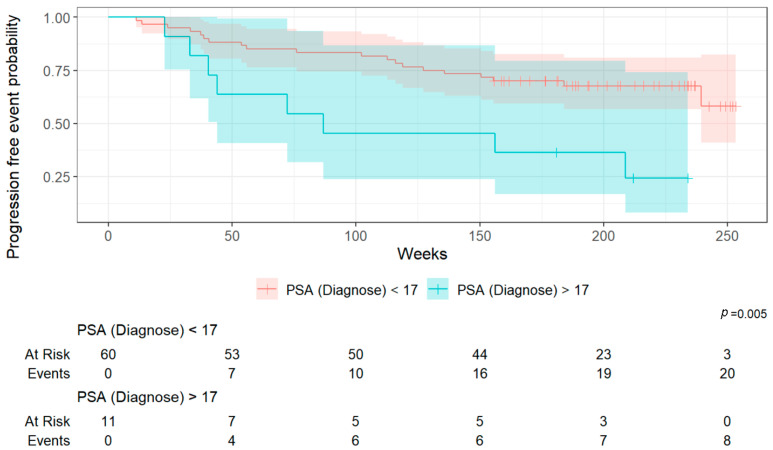
Kaplan–Meier survival curves (PFS) according to PSA value at diagnosis cut-off (17 ng/mL).

**Figure 6:**
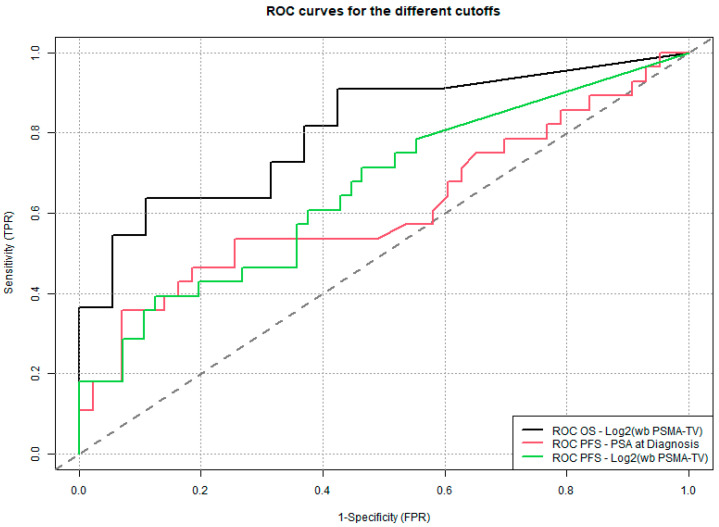
Receiver operating characteristic (ROC) curves comparing the sensitivity and specificity of the different cut-off values. The area under the curve (AUC) in OS-Log_2_(wb PSMA-TV) is 0.81, in PFS-Log_2_(wb PSMA-TV) 0.69, and in PFS-PSA at diagnosis 0.61.

The cut-off value for log_2_(wb TMV) was set at 2.55, while the cut-off value for PSA at diagnosis was determined to be 17 ng/mL. Patients with log_2_(wb TMV) > 2.55 and PSA value at diagnosis of 17 ng/mL had significantly worse survival outcomes compared to those with log_2_(wb TMV) ≤ 2.55 and PSA value at initial diagnosis ≤ 17 ng/mL (Figure 4, Figure 5 and Figure 6).

**Figure 7:**
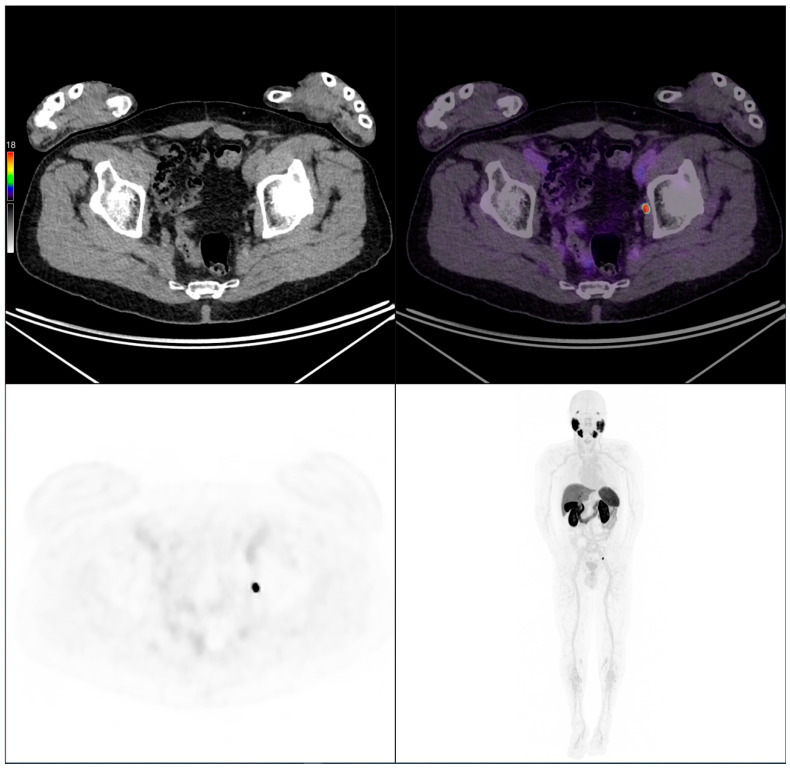
PSMA PET/CT scan of an 83-year-old patient with biochemical recurrence (PSA: 0.4 ng/mL). Initially, the Gleason score was 4 + 3 = 7, pT3 pN0 M0, and treated with radical prostatectomy. Imaging performed following intravenous administration of 208 MBq of [^18^F]PSMA-1007 revealed a left obturator lymph node metastasis consistent with a local recurrence.

**Figure 8:**
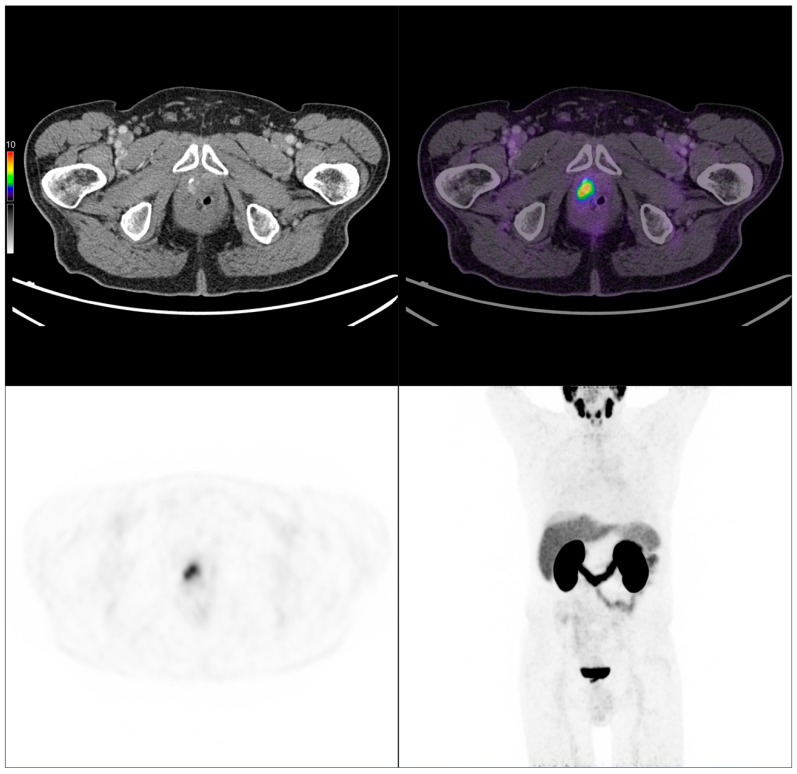
PSMA PET/CT scan of an 84-year-old patient with biochemical recurrence (PSA: 0.67 ng/mL). Initially, the Gleason score was 4 + 3 = 7, pT3 pN0 M0, and treated with radical prostatectomy. Imaging performed following intravenous administration of 141 MBq of [^68^Ga]Ga-PSMA-11 revealed a local recurrence.

To illustrate our findings, two representative clinical cases from the study cohort are presented below, highlighting key imaging features (Figure 7 and Figure 8).

## 4. Discussion

The primary aims of this retrospective study were twofold: first, to compare [^68^Ga]Gallium-68 and [^18^F]Fluorine-18 tracers for PSMA PET/CT imaging in men with biochemical recurrence following definitive treatment of localized prostate cancer, and second, to investigate the predictive value of clinical data and imaging biomarkers based on overall survival and progression-free survival.

Overall, the two patient cohorts (Ga- vs. F-PSMA) showed no significant differences in baseline characteristics or detection rates. In 69% of PSMA-PET/CT scans, a clear imaging correlate for biochemical recurrence was identified, while 17% of scans were classified as indeterminate. Indeterminate bone lesions were more frequently observed with F-PSMA, albeit based on a small sample size. These findings are consistent with previously published studies. For instance, Dietlein et al. and Mingels et al. also reported a higher rate of indeterminate bone lesions using PSA-targeted radioligands labeled with [^18^F]Fluorine, suggesting a potential diagnostic limitation of this tracer [34,35]. Interestingly, a significant difference in OS and PFS was observed between the populations imaged with Ga-68 versus F-18, to the disadvantage of F-18 (52 vs. 38 months, *p* < 0.001). This discrepancy is explained by a shorter follow-up time in the F-18 group, as Ga-68–labeled tracers had been in clinical use for a longer period. Due to increasing demand and logistical considerations, the institution eventually transitioned to F-18.

Importantly, a higher whole-body Total Molecular Volume (wb TMV) was associated with poorer overall survival and progression-free survival. In FDG PET/CT imaging, the predictive value of MTV has thus been validated across various tumor types, such as metastatic melanoma, non-small cell lung cancer, and breast cancer using [^18^F]Fluorine-FDG, where an increased metabolic tumor burden consistently correlates with worse survival outcomes [36,37]. As we have shown previously, the baseline MTV predicted early response to immune checkpoint inhibition in melanoma patients, highlighting the potential of metabolic imaging to guide immunotherapy strategies [38]. Similarly, in non-small cell lung cancer (NSCLC), MTV and metabolic features derived from FDG-PET/CT were associated with clinical benefit and durable responses following immune checkpoint blockade [39,40]. Moreover, our research group developed an innovative non-linear predictive model incorporating baseline FDG-PET/CT metrics and clinical data to predict outcomes in patients with newly diagnosed breast cancer [41].

However, within the context of PSMA PET/CT imaging for prostate cancer, prior studies following the VISION trial primarily focused on the prognostic value of semi-quantitative parameters such as SUVmean [25,26,27]. While these studies have provided important insights into advanced metastatic settings, to the best of our knowledge, no similar published data have yet evaluated the predictive value of whole-body Total Molecular Volume (wb TMV) specifically in the setting of biochemical recurrence following definitive treatment of localized prostate cancer.

This lack of prior research highlights the innovative nature of our findings. By demonstrating that wb TMV quantified from PSMA PET/CT scans can predict survival outcomes even in patients with biochemical recurrence, our study provides novel evidence supporting the use of volumetric tumor assessment as an objective prognostic tool in earlier stages of disease. In a supplementary analysis, we assessed the diagnostic accuracy of the three determined cut-off values for overall survival (OS) and progression-free survival (PFS). Log_2_(wb-PSMA TV) emerged as the most discriminative parameter based on its AUC (0.81), outperforming the two other metrics. However, it is noteworthy that its performance was substantially better for OS than for PFS, suggesting that this parameter may be a more reliable predictor of survival (13% mortality in the overall cohort) than of progression (33% in the overall cohort).

### 4.1. Strengths and Limitations

One of the key strengths of this study lies in its innovative focus on the prognostic value of whole-body Total Molecular Volume (wb TMV) derived from PSMA PET/CT in the setting of biochemical recurrence (BCR) of prostate cancer. While volumetric tumor assessment has been widely explored in advanced disease and FDG PET/CT imaging, its role in the context of BCR remains largely under-investigated. By addressing this gap, our study contributes novel insights into the prognostic utility of volumetric imaging biomarkers in earlier stages of prostate cancer progression.

Furthermore, the inclusion of two radiotracers—[^68^Ga]Ga-PSMA-11 and [^18^F]F-PSMA-1007—enabled a comparative analysis of their diagnostic performance and prognostic relevance, reflecting a real-world clinical transition between tracers and offering practical guidance for imaging workflows.

From a methodological perspective, the manual 3D segmentation of tumor lesions, combined with double-reader validation by dual-certified radiology and nuclear medicine physicians, ensured high internal consistency and robustness of imaging data. The application of standardized thresholds and rigorous statistical methodology—including univariable and multivariable Cox regression models, ROC curve analyses, and cut-off determination using the Youden index—further reinforced the strength of our conclusions. These results may have important implications for risk stratification, treatment planning, and patient selection for subsequent therapeutic interventions, including radioligand therapy.

Despite the promising results of our study, certain limitations must be acknowledged.

First, the retrospective design inherently carries a risk of selection bias, and a number of patients had to be excluded due to missing imaging or incomplete follow-up data. Second, some heterogeneity existed among the included patients, particularly in terms of prior therapies, which may have influenced imaging findings and survival outcomes to a limited extent. Third, the sample size (84 patients) was limited, which could have constrained the statistical power of the survival analyses, especially in multivariable models. Fourth, although double-reading protocols were systematically applied, a certain inter-observer variability in the interpretation of PSMA-PET/CT scans cannot be entirely ruled out. Finally, while the manual segmentation of lesions using structured 3D contouring methods was performed meticulously, the process inevitably involves a certain level of variability, particularly in cases where small lesion boundaries are poorly defined or contrast is limited. Nevertheless, the adoption of standardized procedures and rigorous double-readers (both readers were board-certified in radiology and nuclear medicine) validation at our institution helped minimize this potential bias.

These limitations, while important to consider, do not detract from the innovative nature of our findings. However, to address the limitations inherent to our retrospective, single-center design, several directions for future research should be considered. First, prospective multicenter studies are warranted to increase sample size, reduce potential selection bias, and improve the generalizability of findings. Such studies would allow for broader patient representation and enhance the statistical power of survival analyses.

Second, standardization of imaging protocols across participating centers—including harmonization of tracer selection, acquisition timing, and reconstruction algorithms—would improve data comparability and reduce methodological variability.

Third, the implementation of AI-based or semi-automated segmentation tools represents a promising avenue to reduce inter-observer variability and to facilitate the reproducibility and scalability of volumetric tumor assessment. This could be particularly valuable for widespread clinical adoption.

Additionally, patient stratification based on prior therapeutic modalities and the timing of recurrence would allow for more nuanced interpretation of imaging biomarkers and their prognostic significance.

Finally, extending the follow-up duration in future studies will be essential to capture a greater number of progression and survival events, thereby strengthening multivariable analyses and enabling more detailed subgroup evaluations.

### 4.2. Perspectives and Clinical Implications

Future prospective studies with larger cohorts are warranted to confirm these findings and to further validate the utility of wb TMV quantification for risk stratification and treatment planning, including the selection of candidates for radioligand therapy.

## 5. Conclusions

A higher whole-body Total Molecular Volume (wb TMV) was associated with poorer overall survival and progression-free survival (PFS), supporting its potential role as a prognostic biomarker, particularly for overall survival.

## Figures and Tables

**Table 1:** Descriptive statistics of the study population overall and according to the tracer used.

	Overall	Ga-PSMA	F-PSMA	*p*-Value
	*N* = 84	*N* = 60	*N* = 24	
Age (years)	72 (66–77)	72 (65–75)	75 (69–79)	0.2
BMI (kg/m^2^)	25.2 (23.7–26.6)	24.0 (23.3–26.4)	25.9 (24.6–26.8)	0.2
PSA at initial diagnosis (ng/mL)	8 (6–12)	9 (6–12)	7.0 (6.0–14.0)	0.7
PSA at BCR (ng/mL)	0.6 (0.4–2.2)	0.6 (0.4–2.2)	0.7 (0.4–2.1)	0.6
Gleason Score				0.6
6	4 (4.9%)	3 (5.1%)	1 (4.3%)	
7	35 (43.0%)	28 (47.0%)	7 (30.0%)	
7a (3 + 4)	12 (15.0%)	7 (12.0%)	5 (22.0%)	
7b (4 + 3)	9 (11.0%)	7 (12.0%)	2 (8.7%)	
8	7 (8.5%)	5 (8.5%)	2 (8.7%)	
9	14 (17.0%)	8 (14.0%)	6 (26.0%)	
10	1 (1.2%)	1 (1.7%)	0 (0.0%)	
Not available	2 (1.4%)	1 (1.2%)	1 (1.2%)	
Prior therapy				0.4
Radical prostatectomy	57 (68.0%)	42 (70%)	15 (63%)	
Radical prostatectomy + Salvage radiotherapy	2 (2.4%)	1 (1.7%)	1 (4.2%)	
Radical prostatectomy + Radiotherapy + ADT	11 (13.0%)	9 (15.0%)	2 (8.3%)	
Radiotherapy + ADT	11 (13.0%)	7 (12.0%)	4 (17%)	
Brachytherapy	1 (1.2%)	0 (0.0%)	1 (4.2%)	
Radiotherapy only	2 (2.4%)	1 (1.7%)	1 (4.2%)	
OS (weeks)	198 (170–233)	219 (189–238)	165 (159–184)	<0.001
PFS (weeks)	184 (134–230)	208 (156–234)	159 (115–171)	<0.001
Death	11 (13%)	7 (12%)	4 (17%)	0.7
Progression event	28 (33%)	19 (32%)	9 (38%)	0.6
Observation time (months)	47 (42–54)	52 (45–56)	38 (37–42)	<0.001
wb TL-PSMA	0.03 (0.00–0.19)	0.03 (0.00–0.14)	0.03 (0.00–0.33)	0.3
wb TMV	0.6 (0.0–2.4)	0.6 (0.0–2.2)	0.6 (0.0–6.0)	0.4

BMI = Body Mass Index; PSA = Prostate-Specific Antigen; ADT = Androgen Deprivation Therapy;wb TL-PSMA = whole-body Total Lesion-PSMA; wb TMV = whole-body Total Molecular Volume. OS = Overall Survival; PFS = Progression-Free Survival.

**Table 2:** Pathological PSMAPET/CT findings (Category 1).

	Overall	Ga-68	F-18	*p*-Value
	*N* = 58	*N* = 39	*N* = 19	
Number of correlate(s) for BCR				0.7
1	38 (66%)	26 (67%)	12 (63%)	
2	18 (31%)	11 (28%)	7 (37%)	
3	2 (3.4%)	2 (5.1%)	0 (0%)	
Anatomical site				
Local (Prostatic fossa)	34 (59%)	22 (56%)	12 (63%)	0.6
Pelvic lymph node	22 (38%)	15 (38%)	7 (37%)	>0.9
Retroperitoneal lymph node	12 (21%)	8 (21%)	4 (21%)	>0.9
Supradiaphragmal lymph node	6 (10%)	4 (10%)	2 (11%)	>0.9
Bone	15 (26%)	9 (23%)	6 (32%)	0.5
Soft tissue	0 (0%)	0 (0%)	0 (0%)	
Other	2 (3.4%)	2 (5.1%)	0 (0%)	>0.9

**Table 3:** Indeterminate PSMA PET/CT findings (Category 2).

	Overall	Ga-68	F-18	*p*-Value
	*N* = 14	*N* = 11	*N* = 3	
Local (Prostatic fossa)	4 (29%)	2 (18%)	2 (67%)	0.2
Pelvic lymph node	7 (50%)	7 (64%)	0 (0%)	0.2
Retroperitoneal lymph node	1 (7.1%)	1 (9.1%)	0 (0%)	>0.9
Supradiaphragmal lymph node	1 (7.1%)	1 (9.1%)	0 (0%)	>0.9
Bone	5 (36%)	2 (18%)	3 (100%)	0.027
Soft tissue	0 (0%)	0 (0%)	0 (0%)	
Other	2 (14%)	2 (18%)	0 (0%)	>0.9

**Table 4:** Univariable Cox regression for overall survival (OS).

	HR	95% CI	*p*-Value
wb TL-PSMA	1.24	1.04–1.47	0.018
Log_2_(wb TMV)	2.20	1.55–3.13	<0.001
PSA (initial diagnosis) ng/mL	1.03	0.99–1.08	0.2
PSA (BCR) ng/mL	1.08	1.04–1.12	<0.001
SUVmean	1.00	0.99–1.01	0.8
SUVmax	1.00	0.99–1.01	0.7

HR = Hazard Ratio; CI = Confidence Interval; PSA = Prostate-Specific Antigen; wb TL-PSMA = whole-body Total Lesion-PSMA; wb TMV = whole-body Total Molecular Volume; SUV = Standardized Uptake Value.

**Table 5:** Multivariable Cox regression for overall survival (OS).

	HR	95% CI	*p*-Value
wb TL-PSMA	1.03	0.76–1.40	0.8
Log_2_(wb TMV)	1.94	0.98–3.84	0.057
PSA (BCR) (ng/mL)	1.01	0.95–1.08	0.7

HR = Hazard Ratio; CI = Confidence Interval; PSA = Prostate-Specific Antigen; wb TL-PSMA = whole-body Total Lesion-PSMA; wb TMV = whole-body Total Molecular Volume.

**Table 6:** Univariable Cox regression for progression-free survival (PFS).

	HR	95% CI	*p*-Value
wb TL-PSMA	1.14	0.98–1.32	0.10
Log_2_(wb TMV)	2.01	1.46–2.77	<0.001
PSA (initial diagnosis) ng/mL	1.04	1.01–1.07	0.006
PSA (BCR) ng/mL	1.04	1.00–1.08	0.037
SUVmean	1.00	0.99–1.01	0.6
SUVmax	1.00	0.99–1.01	0.5

HR = Hazard Ratio; CI = Confidence Interval; PSA = Prostate-Specific Antigen; wb TL-PSMA = whole-body Total Lesion-PSMA; wb TMV = whole-body Total Molecular Volume. SUV = Standardized Uptake Value.

**Table 7:** Multivariable Cox regression for progression-free survival (PFS).

	HR	95% CI	*p*-Value
Log_2_(wb TMV)	1.78	1.19–2.65	0.005
PSA (initial diagnosis) ng/mL	1.04	1.00–1.07	0.036
PSA (BCR) ng/mL	1.01	0.95–1.07	0.8

HR = Hazard Ratio; CI = Confidence Interval; PSA = Prostate-Specific Antigen; wb TL-PSMA = whole-body Total Lesion-PSMA; wb TMV = whole-body Total Molecular Volume.

## Data Availability

All reviewed imaging modalities and clinical data were assessed during clinical routine. Patient data are stored in a local archiving system.

## Abbreviations

ADT	Androgen Deprivation Therapy
BCR	Biochemical Recurrence
BMI	Body Mass Index
CRPC	Castration-Resistant Prostate Cancer
CT	Computed Tomography
MBq	Megabecquerel
MTV	Metabolic Tumor Volume
OS	Overall Survival
PET	Positron Emission Tomography
PFS	Progression-Free Survival
PSA	Prostate-Specific Antigen
PSMA	Prostate-Specific Membrane Antigen
RLT	Radioligand Therapy
ROC	Receiver Operating Characteristic
ROI	Region Of Interest
SUV	Standardized Uptake Value
wbTMV	whole-body Total Molecular Volume
wbTL-PSMA	whole-body Total Lesion PSMA
